# Temporal and Spatial Trends in Childhood Asthma-Related Hospitalizations in Belo Horizonte, Minas Gerais, Brazil and Their Association with Social Vulnerability

**DOI:** 10.3390/ijerph13070704

**Published:** 2016-07-12

**Authors:** Cláudia Silva Dias, Maria Angélica Salles Dias, Amélia Augusta de Lima Friche, Maria Cristina de Mattos Almeida, Thaís Claudino Viana, Sueli Aparecida Mingoti, Waleska Teixeira Caiaffa

**Affiliations:** 1 Department Physiotherapy, Pontifical Catholic University of Minas Gerais (PUC Minas), Belo Horizonte 30535610, Brazil; 2 Belo Horizonte Observatory for Urban Health (OSUBH), School of Medicine, Federal University of Minas Gerais (UFMG), Belo Horizonte 30130100, Brazil; angelica@pbh.gov.br (M.A.S.D.); gutafriche@gmail.com (A.A.L.F.); cristinaalmeida@gmail.com (M.C.M.A.); thais_tcv@hotmail.com (T.C.V.); suelimngt@gmail.com (S.A.M.); wcaiaffa@medicina.ufmg.br (W.T.C.); 3 Institute of Exact Sciences, Federal University of Minas Gerais (UFMG), Belo Horizonte 31270901, Brazil

**Keywords:** hospitalization for asthma, ecological study, vulnerability, children and adolescents

## Abstract

*Introduction*: Asthma is a multifactorial disease and a serious public health problem. Environmental factors and poverty are the main determinants of this disease. *Objective*: To describe the spatial and temporal distribution of asthma-related hospitalizations and identify the areas with the highest prevalence of and vulnerability to severe asthma in a major Brazilian city. *Methods*: An ecological study of hospitalizations for asthma from 2002 to 2012, in children and adolescents under 15 years of age, living in Belo Horizonte, Southeast Brazil. All events were geocoded by residence address using Hospital Information System data. The socioeconomic vulnerability of residence address was ranked using the Health Vulnerability Index. Raster surfaces were generated and time-series plots were constructed to determine spatial and time trends in the frequency of asthma-related hospitalizations, respectively. *Results*: Asthma-related hospitalization rates were highest in children aged 0–4 years and in boys. There was a decreasing trend in the number of asthma-related hospitalizations across the study period. Approximately 48% of all hospitalizations were children living in health vulnerable areas. Seasonal trends showed a hospitalization peak in March, April, and May, coinciding with the post-rainy period. *Conclusion*: Our findings suggest that social and environmental factors may be determinants of disparities in severe asthma.

## 1. Introduction

Asthma is a chronic inflammatory disease of the airways in which many cells and cellular elements play a role. It is associated with lower airway hyper-responsiveness and airflow obstruction that are reversible spontaneously or with treatment. Typical respiratory symptoms include wheezing, shortness of breath, chest tightness and coughing, particularly at night or early in the morning [1].

Moreover, asthma is the most common chronic disease in children and adolescents, resulting in high health care costs, school absenteeism and parental absenteeism from work, as well as reduced quality of life, reflected in both poor academic performance and social interaction [2,3].

Asthma is a global health problem that affects an estimated 300 million individuals worldwide [1]. In Brazil, over 20 million people are affected by asthma, considering a global prevalence of 10% of the population [4,5], where one in four preschool children is affected by asthma [5]. The average prevalence of asthma among Brazilian children and adolescents has been estimated at 23.8% in boys, 20.4% in girls aged 6–7 years, 19% in 13–14-year-old adolescents in São Paulo [5]. A prevalence of 17% in adolescents from 13 to 14 years old was found in Belo Horizonte [6].

Asthma has increased in children living in urban areas and in poverty [7,8]. With the projected increase in the proportion of the world’s urban population from 45% to 59% in 2025, together with an increased proportion living in poor urban areas, it is likely to have a significant increase in the number of asthmatics worldwide, over the next two decades. It is estimated that there may be an additional 100 million people with asthma by 2025 [1]. 

It is well known that urban environment components that include poor housing conditions are associated with increased exposure to indoor allergens, residential segregation, physical environmental risk, psychological stress, caregivers’ attitudes toward less use of asthma medications and poor access to health care services. All of them may contribute to asthma disparities [8,9] and case aggravations.

The number of hospital admissions due to asthma is an indicator of poor control of the disease and asthma is the second leading cause of hospital admissions in children under 14 years of age. Despite the fact that the rate of asthma hospitalization has been decreasing in Brazil, in Minas Gerais and in its capital, Belo Horizonte City [10], high primary admission and readmission rates (112.8 and 25.0 per 10,000 children aged 0–5 years in 1997 and 2000, respectively) have been observed [11].

Severe cases with exacerbated signs and symptoms that lead to hospitalization and, in some cases, death, result from inadequate self-management by the patient and a failure of the health systems to provide appropriate care [1,12]. Appropriate asthma management, preferably in primary care units such as access to preventive medication, family and patient guidelines about the care that should be taken with the disease and staff training for disease management capacity can improve the quality of life as well as prevent unnecessary emergency department visits and avoidable hospitalizations [13,14].

The seasonal variation in asthma cases, especially the poor control of cases, is another important reason for the occurrence of asthma. Studies in different regions of the world have found significant associations between hospital admissions for asthma and seasonality [15,16]. Nevertheless, evidence of this association and the influence of physical and chemical environmental variables on asthma prevalence remain scarce. 

This study aimed to describe and analyze the spatial and temporal distribution of the frequency of asthma-related hospitalizations among children and adolescents living in an urban area, identifying areas with highest prevalence of and social vulnerability to poor control of the disease. The greatest contribution of this study may be to furnish the debate that should be given to this disease to prioritizing distal interventions and investments by public policies [17], in order to contribute to the disease prevention and promotion. 

## 2. Methods

This is a descriptive and ecological study of the asthma-related hospitalization frequency (ARHF) and asthma-related hospitalization rates (ARHR). The study period was from 1 January 2002 to 31 December 2012, where it included children and adolescents aged 0–14 years living in Belo Horizonte (BH) and admitted to public and/or agreement hospitals of the Belo Horizonte Unified Health System (SUS-BH). BH currently has 5302 SUS-BH hospital beds, 674 of which are pediatric ones.

The city of Belo Horizonte (19.9° S and 43.9° W, 900 m average elevation) is the capital of the Brazilian state of Minas Gerais. It is a city with over 2.3 million habitants, located in the southeast of Brazil, where 27% of this population lives in areas of high poverty [7]. The city extends over an area of 331.4 square km^2^, with a population density of 7167.02 inhabitants by square km^2^ [18]. The climate is tropical with an annual average temperature of approximately 21 °C and annual average precipitation higher than 1300 mm. Summer extends from January to March; Autumn months are April, May, and June; Winter is during July, August, and September; and Spring is during October, November, and December.

### 2.1. Study Variables

All hospital admission authorizations for asthma in children and adolescents aged 0–14 years, from all public and agreement SUS-BH hospitals stored in the Unified Health System Hospital Information System (SIH/SUS) database between 2002 and 2012 and provided by the Belo Horizonte Municipal Health Department (SMSA/PBH), using International Classification of Diseases, Tenth Revision, Clinical Modification (ICD-10-CM) diagnosis codes (J45.0 to J46), were used as the response variable. The explanatory variables were composed of information blocks regarding the patient (gender, age, date of birth), residence (street, number, and neighborhood), admission (year and month of hospitalization, number of days of stay), and deaths. Data were collected on a yearly basis from 2002 to 2012.

### 2.2. Geocoding

The census tract was used as the spatial unit, whereas the year and month of asthma admissions were used as the temporal unit. The geographic database of census tracts for 2010 was provided by SMSA/PBH, as defined by the Brazilian Institute of Geography and Statistics (IBGE) [18].

Each asthma hospitalization event was geocoded by residence address vector coordinates (x,y) taken from the residence street and number using a geocoding system (SISGEOW) of the Belo Horizonte street dataset. If the exact residence address could not be geocoded, the event was matched to an approximate address in the same street (±100 street numbers). If previous attempts failed and the street had only one block, the address was geocoded to the street centroid. Each geocoded event was matched to its respective census tract classified according to the Health Vulnerability Index (HVI). 

#### The Health Vulnerability Index (HVI)

The HVI is an index composed of a combination of social, demographic and economic indicators, developed by the SMSA/PBH. The HVI was determined in 1998, recalculated in 2003 using the 2000 census data, updated and adjusted in 2012 using the 2010 census data. Based on the selection of spatial inequalities’ indicators and on the analyses by means of the participatory method [7], weights, by Delphi method were given to each indicator, where the higher the value, the less favorable is the situation of the population.

The following indicators and assigned weights were used for the calculations: (A) sanitation (inadequate water supply 0.424, inadequate sanitary sewage 0.375 and inadequate garbage collection 0.201); (B) housing (residents per household 0.073); (C) education (illiterate population 0.283); (D) income (per capita income up to ½ minimum wage 0.288, responsible people’s average income 0.173); (E) social (black and indigenous people’s percentage 0.185) [7].

Based on the registered values of all census tracts, the following cutoff points were established for the HVI: (A) Medium Risk—census tracts with ½ standard deviation HVI values around the mean (mean +/− SD 0.5). (B) Low risk—tracts with HVI values lower than mean HVI. (C) High risk—tracts with values above mean HVI, up to 1.5 standard deviation above the average limit (upper limit of the average HVI + 1 SD). (D) Very high risk—tracts, with values above the high HVI. In the present study, data for the high and very high vulnerability areas were grouped together because of the small number of children living in very high vulnerability areas.

According to our conceptualized framework on asthma determination, the HVI, a GIS (geographical information system) composite indicator, would be able to provide a better understanding of the socio-economic and environmental context in which the hospitalized asthma patient lived given the well-known influence of neighborhood disadvantage on several children's health outcome due to the sanitary conditions, physical infrastructure and economic as well as social status [7].

### 2.3. Time Series

The frequency of monthly hospitalizations over the study period was plotted using time-series plots.

## 3. Statistical Analysis

### 3.1. Hospitalization Rates

Asthma-related hospitalization rates in each year were calculated from the age-group-matched number of hospitalizations and city population. The number was estimated on a yearly basis and considering the need for demographic reconciliation resulting from the omission of individuals at the time of population enumeration in the 2010 IBGE Census. The omission rate for boys and girls aged 0–4 years was 15% and 16%, respectively, and 13% for the 5–9 year age group. Thus, a single correction factor of 14.6% was applied to these two age groups [18].

The average annual growth rate for the city was calculated based on population changes in specific areas such as formal areas and slums [19]. The 2010 base population was estimated from the 2000 population and used to calculate the cohort growth rate for the city in the intercensal period [19].

Differences in hospitalization rates across age groups (0–4, 5–9, and 10–14 year age groups) and between genders from 2002 to 2012 were compared using analysis of variance (ANOVA) and the Student’s *t* test, respectively. Pairwise comparisons among age groups were performed using the Bonferroni test [20]. Differences were considered significant at *p* < 0.05.

Yearly differences in the number of hospitalizations across vulnerability levels were compared using analysis of variance (ANOVA) and pairwise comparisons between levels were performed using the Bonferroni test. Differences were considered significant at *p* ≤ 0.05 [20].

The hospitalization rates for the years 2002 and 2012 were calculated for each HVI category (low, medium, high/very high). The number of hospital admissions for asthma in the respective years was considered in the numerator and the population count of the 2000 and 2010 census in the denominator [18,21]. The Risk Ratio (RR) and 95% confidence intervals were then calculated stratified by HVI in order to compare the 2002 and 2012 hospitalization rates.

### 3.2. Spatial and Time Trends

Kernel maps of the frequency of asthma-related hospitalization were generated to determine the spatial distribution of hospitalizations in the city of BH [22].

A time-series plot with seasonal decomposition was constructed for the analysis of time trends in asthma-related hospitalizations [23].

The descriptive analysis was done using Statistical Package for the Social Sciences (SPSS^®^) version 22.0 (IBM Corp.: Armonk, NY, USA). Residence addresses were geocoded using SISGEOW software (Municipal Health Department, Belo Horizonte, Minas Gerais, Brazil). Kernel analysis was carried out using CrimeStat (Ned Levine & Associates, Houston, TX, USA and the National Institute of Justice, Washington, DC, USA) and maps were constructed using MapInfo 10 (MapInfo Corporation, North Greenbush, NY, USA), whereas the time series analysis was conducted using R software (Free Software Foundation’s GNU project, Auckland, New Zealand). This study was part of a project named the Health of Residents in Special Areas of Social Interest and was approved by the research ethics committee at the Health Department of Belo Horizonte Municipality and Federal University of Minas Gerais under the respective protocol number CAAE 11548913.3.0000.5149. 

## 4. Results

### 4.1. Hospitalization Rates

**Figure 1:**
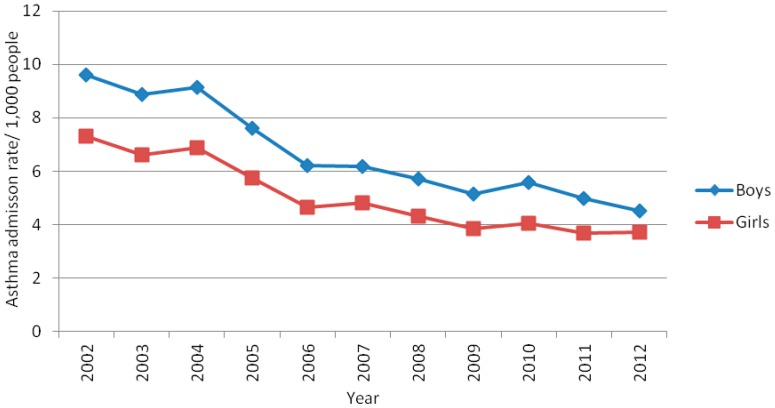
Rate of asthma-related hospitalizations in children and adolescents aged 0–14 years in Belo Horizonte, Minas Gerais, Brazil, by gender, 2002–2012.

In Belo Horizonte, there were 36.975 asthma-related hospitalizations from 2002 to 2012 across all age groups, 89% of which were among children aged 0 to 14 years, totalizing 32,978 childhood hospitalizations (Table 1). The number of hospitalizations was highest in the 0–4 year age group across years and 35% higher in boys than in girls across age groups (Table 1). Moreover, hospitalization rates were higher in boys than in girls across years (Figure 1).

**Figure 2:**
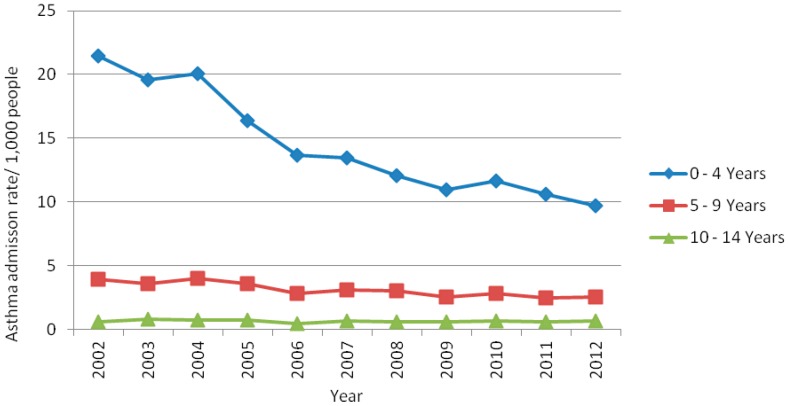
Asthma-related hospitalization rates in children and adolescents aged 0–14 years in Belo Horizonte, Minas Gerais, Brazil, by age group, 2002–2012.

Asthma-related hospitalization rates were significantly different (*p* ≤ 0.05) across age groups and there was a decreasing trend in hospitalization rates over the 11-year study period. Specifically, hospitalization frequency declined by approximately 55% and 36% in the 0–4 and 5–9 year age groups, respectively. Conversely, ARHR in the 10–14 year age group were stable from 2002 to 2012 (Figure 2).

The average length of hospital stay among children aged 0 to 14 years was three days; 10 deaths were registered, all in the 0–4 year age group, eight of them were residents in informal settlements, also named as slums.

### 4.2. Spatial Trends

Of the 32,978 childhood asthma-related hospitalizations, 21,758 (66%) were geocoded to the exact address and 8009 (24%) to the approximate address, whereas in 10% of the hospitalizations the home address could not be geocoded.

**Figure 3:**
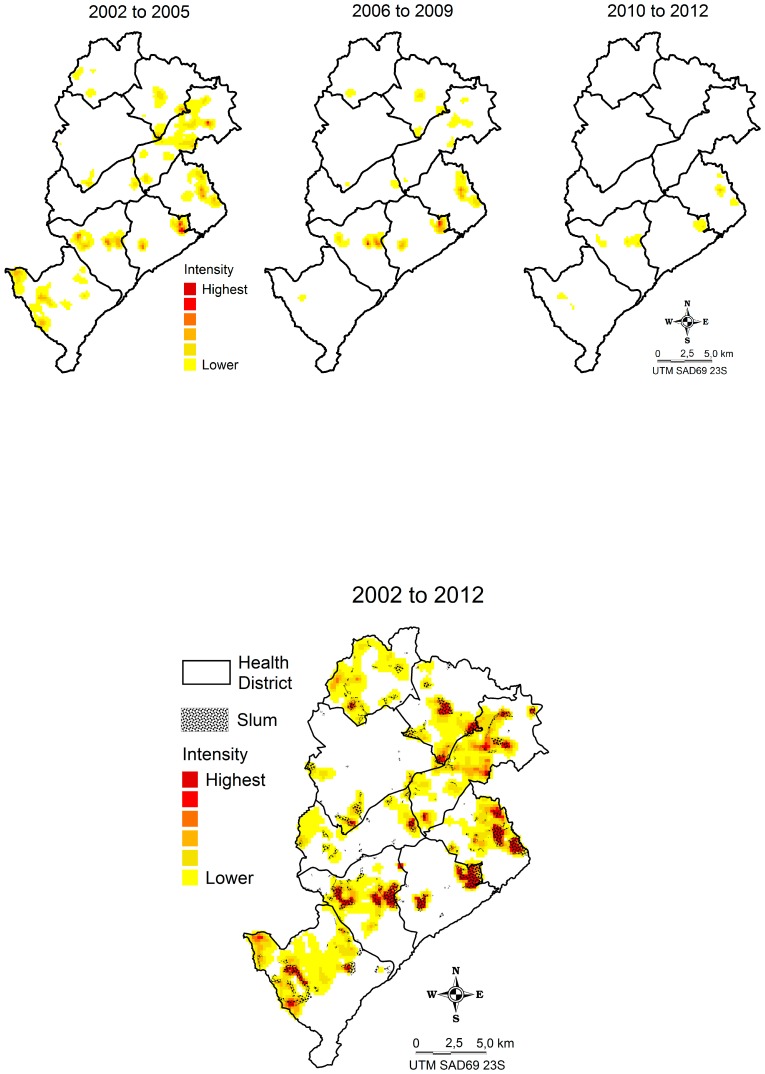
Frequency of asthma-related hospitalization in children and adolescents aged 0–14 years in Belo Horizonte, Minas Gerais, Brazil, by residence address, 2002–2012.

The Kernel map shows that hospitalization frequency was highest in slums (“hotspots”). In the figure below, slums are highlighted in yellow in each of the nine health districts (these are geographic and administrative areas in the city) over the study period (Figure 3). 

**Figure 4:**
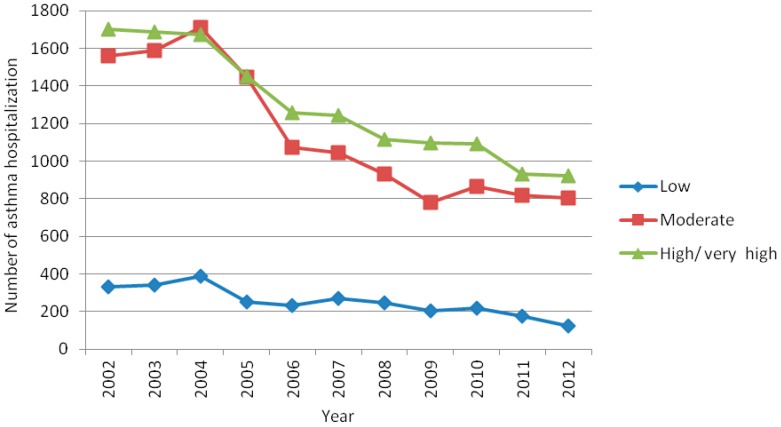
Frequency of asthma-related hospitalizations in children and adolescents aged 0–14 years in Belo Horizonte, Minas Gerais, Brazil, by social vulnerability level, 2002–2012.

Of the 29,767 geocoded cases, 29,570 were matched to the home address according to the HVI census tract classification, 14,170 (48%) hospitalizations occurred in high/very high-risk HVI census tract, 12,619 in medium-risk (43%), and 2,781 (9%) in low-risk HVI census tract. Thus, approximately 91% of all asthma-related hospitalizations were children living in moderate and high/very high-risk areas, represented herein by the cluster of the census tract by HVI classification (Figure 4). In fact, the hospitalization numbers were significantly higher in moderate and high/very high-risk areas than in low-risk areas, when compared according to the year (ANOVA = 95%, *p* ≤ 0.05).

The hospitalization rates for asthma in children and adolescents for the low, medium and high/very high risk areas were, respectively, 3.4, 12.5 and 19.6 per 1000 people, in 2002. In 2012, they were 1.7, 9.0 and 15.3, respectively. 

**Figure 5:**
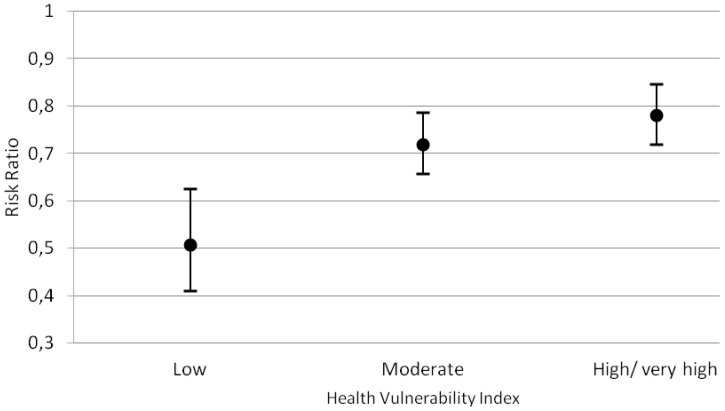
Stratified Risk Ratio of asthma-related hospitalization rates in children/adolescents, by social vulnerability level, in Belo Horizonte, Minas Gerais, Brazil, 2002–2012.

Comparing to the rates in 2002 the hospitalization ratio for children/adolescents living in HVI low risk areas reduced by 50% in 2012 (RR: 0.50; 95% CI 0.40 to 0.63). For the medium HVI and high/very high risk areas the reduction was 28% (RR: 0.72; 95% CI 0.66 to 0.79) and 22% respectively (RR: 0.78; 95% CI 0.72 to 0.85) (Figure 5). 

### 4.3. Time Trends

**Figure 6:**
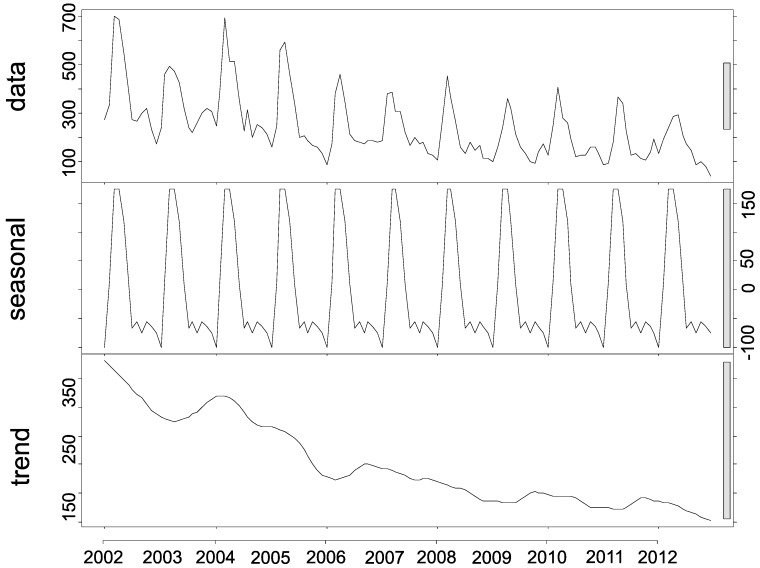
Seasonal decomposition of frequency of asthma hospitalizations in children and adolescents aged 0–14 years in Belo Horizonte, Minas Gerais, Brazil, 2002–2012.

The seasonal decomposition of asthma-related hospitalizations highlighted their seasonal trend and decrease, but non-significant trend, in hospitalizations over the 11-year study period, indicating that the time series is stationary (Figure 6). Additionally, the seasonal variation profile for asthma-related hospitalizations showed distinct peaks in the first semester of each year, indicating a non-random pattern over the 11-year study period, in which each month was represented in a series (Figure 6).

**Figure 7:**
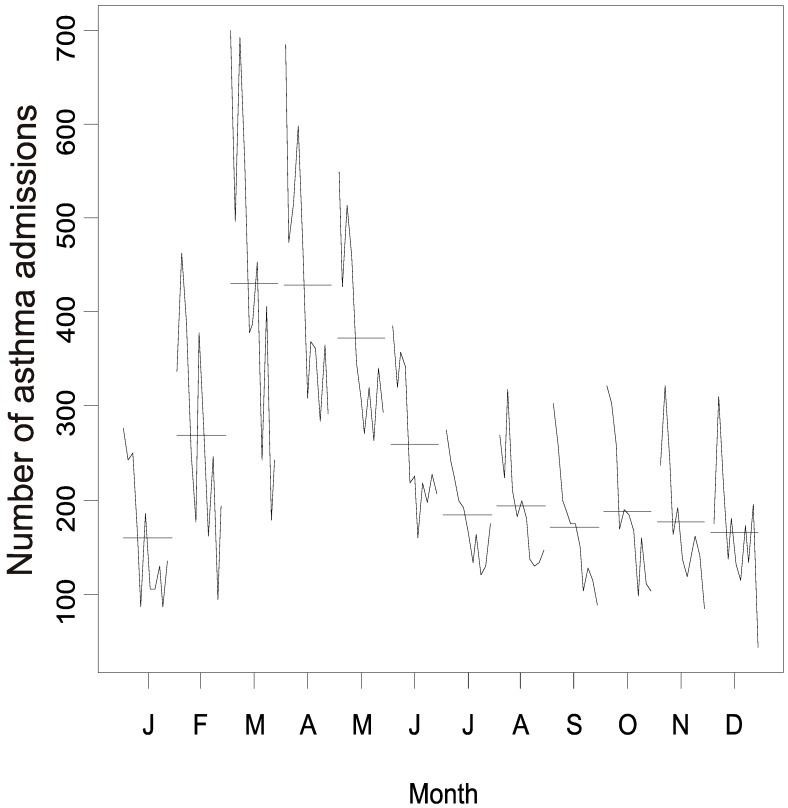
Seasonal subseries plot of asthma hospitalizations in children and adolescents aged 0–14 years in Belo Horizonte, Minas Gerais, Brazil, 2002–2012.

There was an increasing trend in the number of hospitalizations in the same months during the 11-year study period, with an increase in February, followed by a peak in March, April, and May and a decreasing trend from June onwards for all study years, resulting in epidemic episodes in summer and autumn (Figure 7).

## 5. Discussion

The main findings of this study regarding the ARHR among children and adolescents aged 0–14 years in Belo Horizonte from 2002 to 2012 were: (a) the rates were the highest in both the 0–4 year age group and in boys; (b) the rates decreased considerably over the 11-year study period; (c) the frequencies were higher in areas of greater social vulnerability; and (d) the frequencies were markedly seasonal.

### 5.1. Asthma-Related Hospitalization and Rates

Similarly to this study, the *International Study of Asthma and Allergies in Childhood* (ISAAC) also reported a high prevalence of asthma among children and adolescents aged 0–14 years in Brazil [5,24]. Moreover, high hospitalization rates in boys aged 0–4 years and a decreasing trend in hospitalizations have also been reported elsewhere [25]. Also corroborating with our findings, the Brazilian Ministry of Health (MS) reported that ARHR declined by 49% in people ≥ 20 years of age [4] and a study observed a decline of 31.8% in people < 20 years from 2000 to 2010 [26].

Several factors may have contributed to the reduction in ARHR observed in the study. The increased use of preventive medication, especially inhaled corticosteroids, and improved primary diagnosis combined with early interventions [1] indicate that action plans undertaken by health authorities can significantly reduce the number of severe asthma cases and related hospitalizations. In BH, one such action is the Wheezing Child Program [27] established by SMSA/PBH and the Federal University of Minas Gerais in 1996, which prioritizes children aged 0 to 14 years with asthma symptoms. This program aims to improve patient access to preventive medication, monitors children with severe or poorly managed asthma, and provides treatment guidance to patients’ families. Moreover, actions such as expanding prenatal care, encouraging breastfeeding, as well as implementing and expanding Family Health Teams (FHT) may also improve health care access for children and adolescents [28].

Until the year 2000, Belo Horizonte had basic health units that followed SMSA/PBH health care guidelines. Since 2002, the Family Health Program (FHP) has been restructuring the basic health network to incorporate health vulnerability [28,29] equipping the health facilities and increasing the number of FHT in vulnerable areas.

Since 2003, FHT have been restructuring the health care services provided at basic health units to include childhood asthma care, resulting in a 25% increase in the number of consultations, and broadening the practice scope of general practitioners, who accounted for 45% of all consultations for asthma patients in the city over the period across all age groups [28].

This study corroborates the findings of the study done by Bastos et al. [10] that also reported a reduction in asthma hospitalization rate in Minas Gerais (2000–2010). However, the authors did not attribute the drop in hospitalization to the FHT actions, but rather to many factors including intersectoral actions, which turns the association between team actions and outcomes difficult. Besides, the increased FHT coverage and the Wheezing Child Program actions, Belo Horizonte is also implementing slum upgrading policies, youth and adults inclusion in the schools among other policies that may have impacted in improving the care of this disease.

Despite the interventions aimed at improving asthma control in vulnerable populations, the strategies adopted do not seem to be effective and the poor control of the disease may be shaped by the socioeconomic and environmental context [30].

### 5.2. Spatial Trends

The largest concentrations of severe or poorly controlled asthma cases were observed in the most vulnerable areas of the city, such as slums (Figure 3), suggesting that the multiple causes of asthma and the influence of the socioeconomic context [8,9] may play a significant role in this type of health event. Similar findings were observed in genetically similar populations, which showed large variations in the prevalence of asthma, suggesting that environmental factors may play a significant role in determining asthma prevalence [31,32].

Like other Brazilian cities, Belo Horizonte has unequal income distribution and poverty pockets. Thus, the association of asthma hospitalizations with a socioeconomic proxy (i.e., the HVI) within the urban space can help identify and study population groups at the risk for asthma who are living in certain geographic areas of the city, as well as the social determinants of this disease.

Frequency and the rate of asthma-related hospitalizations were highest in high/very high social vulnerability areas, but other contributing factors may have played a role in the occurrence of the poor control of asthma. Household crowding, psychological stress, susceptibility to viral and parasitic infections, housing quality and neighborhood context [8] and exposure to violence [33], have also been associated with increased allergen levels, stress and behavioral factors, to which socially vulnerable children are particularly susceptible.

The role of socioeconomic vulnerability in determining asthma disparities has long been noted. New studies, exploring the indoor and outdoor environmental factors are necessary to understand better the persistence of asthma in geographic areas of greater social vulnerability, especially in cities where health care access policies have been implemented. In addition, studies to assessing the patients’ adherence to treatments are welcomed in order to further understand the increment of the cases [34].

### 5.3. Time Trends 

The seasonal pattern of asthma hospitalizations in this 11-year time series suggests that weather conditions may have played a role in asthma prevalence among children in Belo Horizonte. Changes in weather conditions are known to trigger asthma symptoms, increase the probability of severe allergy attacks in sensitized patients and contribute to exacerbations that may lead to hospitalization [35].

In this study, seasonal trends showed a hospitalization peak in March, April and May for all the studied years, which corresponds to the post-rainy period in the city [36]. This weather condition combined with higher wind speeds in the period increased indoor humidity and triggered fungal spore production and dissemination [37].

The seasonality in adult asthma admissions and the associated seasonality in levels of air pollutants and climatic factors were examined during a four-year period (1998–2001) in Taiwan [38]. The authors reported that adult asthma admissions correlated negatively with temperature (r = −0.76) and hours of sunshine (r = −0.56) and that admission rates increased by 28% in March, which is characterized by increased rainfall in the island.

In the present study the highest number of hospitalizations, especially during the post rain periods, in the city and in socially more vulnerable areas, can be attributed to the increase in indoor humidity with the presence of mold and the risk of viral spread by agglomeration, which put the worst quality homes in greater structural disadvantages. Studies based on population surveys may further clarify these findings and help develop strategies to prevent and/or control of topic and atopic diseases.

### 5.4. Limitations

The study has some limitations related to the design, data sources, use of Health Unify System hospitalization records only and exclusion of socio-demographic data such as income and race/ethnicity. 

Regarding race and ethnicity, a note of caution should be mention in regarding the occurrence of hospital admissions for asthma in specific ethnic and racial groups. It must be emphasized the strong association with these variables with others such as socioeconomic status, access to services, education and marital status. Even not focus of this study we must recommend further studies in this field [39].

To calculate hospitalization rates, corrections of yearly population estimates for children under nine years of age were carried out as recommended by IBGE [18]. Nevertheless, demographic analyses confirmed the decreasing trend in fertility rates and population size (10%) for the 0 to 14 year age group projected for the country in the 2000 census [18]. Thus, the sharp reduction in hospitalization rate observed in this study cannot be explained by the population decline in this age group.

It should be noted that even though this is a descriptive study based on secondary data, which are prone to information bias, hospital information systems are currently the most useful tools in public health and have improved the quality of the information they generate. Moreover, the findings of the study are in agreement with the literature, lending further support to the quality of information [40]. 

The inclusion of public hospitalizations only in our study implies that a portion of the population was excluded from the analyses. Nevertheless, according to the 2013 National Health Survey, ⅔ of the Brazilian population were admitted to public hospitals, especially persons aged < 17 years old [41], thus, the study was able to infer its findings to a large portion of the population.

The study did not investigate allergic and non-allergic asthmas separately, since 97% of the admissions were classified as unspecified asthma. The suggestion is that new studies should analyze hospitalizations for both viral and atopic asthmas in the city. 

Finally, some symptoms of asthma in children under two years of age might not be exclusive to asthma, as the bronchiolitis symptoms for instance [1]. Further studies should be carried out taking into account a possible bias of diagnosis. However, a recent Brazilian study observed that hospitalization for asthma ranked third among all causes of hospitalization among children from 28 days to 1 year of age [26].

## 6. Conclusions

The main contribution of this study is the ability to identify geographic areas at different risk levels for asthma hospitalization, enabling the public health system to manage asthma according to the specific demands of each territory by considering the social and environmental context as determinants for asthma disparities.

Moreover, understanding the local seasonal trends and environmental triggers may help reduce asthma hospitalizations and improve control of the disease. However, it is still unclear whether asthma exacerbations are affected by climatic factors directly or indirectly through their effects on other environmental factors and individual behavior.

## Figures and Tables

**Table 1:** Frequency of asthma hospitalizations by age group in children and adolescents aged 0–14 years from 2002 to 2012 in Belo Horizonte, Minas Gerais, Brazil.

Admission Year	Age Group						Total	Admission Rate/1000 Persons
	0–4		5–9		10–14			
	N	%	N	%	N	%		
2002	3716	82.43	683	15.15	109	2.42	4508	8.47
2003	3326	81.54	611	14.98	142	3.48	4079	7.75
2004	3366	80.62	682	16.34	127	3.04	4175	8.02
2005	2699	78.41	613	17.81	130	3.78	3442	6.68
2006	2214	79.64	480	17.27	86	3.09	2780	5.45
2007	2148	77.16	525	18.86	111	3.99	2784	5.52
2008	1893	75.69	506	20.23	102	4.08	2501	5.01
2009	1699	76.29	423	18.99	105	4.71	2227	4.51
2010	1773	75.13	469	19.87	118	5.00	2360	4.82
2011	1612	76.11	408	19.26	98	4.63	2118	4.34
2012	1480	73.85	417	20.81	107	5.34	2004	4.05
Total	25,926	78.62	5,817	17.64	1235	3.74	32,978	8.47
